# Think Beyond the Room: Measuring Relative Humidity in the Home Cage and Its Impact on Reproduction in Laboratory Mice, *Mus musculus*

**DOI:** 10.3390/ani14223164

**Published:** 2024-11-05

**Authors:** Amanda J. Barabas, Ronald A. Conlon, Craig A. Hodges

**Affiliations:** Department of Genetics and Genome Sciences, Case Western Reserve University, Cleveland, OH 44106, USA; rac14@case.edu (R.A.C.); cah2@case.edu (C.A.H.)

**Keywords:** relative humidity, extrinsic factor, laboratory mouse, animal welfare, mouse reproduction, animal husbandry

## Abstract

Relative humidity (RH) is measured in vivaria with a broad range to accommodate seasonal fluctuations. It is assumed that measurements in the room (macroenvironment) reflect those in the cage (microenvironment). However, there is limited data comparing RH in the macroenvironment to the microenvironment and how the mice may be affected by variations in RH that fall within husbandry recommendations. This study aimed to compare RH in the macroenvironment to that of the microenvironment in various group sizes of laboratory mice; and examine how variation in microenvironmental RH impacts pup survival. Temperature and RH were measured using a temperature/humidity data logger attached to a solid top cage lid. The lid was rotated across *N* = 48 breeding trios and *N* = 33 same sex cages on a C57BL/6J background. Further, once a week, a single breeding trio was selected (*N* = 23) to compare RH readings to weekly rates of pup loss in a larger breeding colony. Across all cages, RH was higher in the microenvironment than the macroenvironment. RH was universally higher in the summer than in the winter, and increased with group size. For breeding cages, as microenvironmental RH increased, the proportion of pups lost each week decreased in a linear relationship. No threshold of decreased mortality could be identified. These data highlight RH as a potential extrinsic factor. While these patterns are correlational, they warrant further research focused on the causative role of RH on mouse welfare.

## Simple Summary

It is standard practice to record relative humidity in vivarium housing rooms. This reflects the animals’ macroenvironment, which should not be assumed to reflect the microenvironment of rodents’ ventilated cages. The aim of this study was to compare relative humidity in the housing room to that inside of ventilated cages. Data were collected across multiple seasons, and groups sizes in both same sex cages and breeding trios. Weekly humidity data were also compared to weekly rates of pup loss in the breeding cages. Overall, humidity was higher inside the home cage (microenvironment) than the housing room (macroenvironment). Humidity was also higher in the summer than in the winter, and when there were more mice in the cage. Further, there was an inverse linear correlation between home cage humidity and pup loss: pup loss increased when humidity was lower. These data show that relative humidity is not always well controlled in the vivarium, and displays seasonal variation. Relative humidity is also highlighted as a potential extrinsic factor affecting animal welfare. However, further work is needed to understand the full scope of relative humidity’s impact on mouse physiology.

## 1. Introduction

Extrinsic factors in animal facilities have been recently emphasized for their impacts on animal welfare and the quality of preclinical research data. Parameters that are not often recorded, such as ambient sound, vibration, and light can have unintended effects on laboratory mice, and it is suggested that they are monitored and reported in the literature [1,2,3]. Furthermore, even parameters that are routinely recorded are not often considered for their impact on animal models. For instance, temperature and relative humidity (RH) are routinely monitored in animal housing rooms as they should be controlled per The Guide for the Care and Use of Laboratory Animals [4]. However, these records are typically based on a single data logger placed in the open air of rodent housing rooms, even though individually ventilated caging (IVC) systems are prevalent in modern animal facilities. In essence, the temperature and RH data that are collected and often reported in publications reflect the macroenvironment of the housing room, whereas the animals are contained in their own microenvironments.

Past work has demonstrated that both temperature and RH are higher in occupied mouse cages than in the animal room [5,6]. However, this work is based on a limited population in that all of the animal subjects were female, of a single strain, and housed in groups of five. More notably though, these data were obtained when the RH in the housing room was within the guide’s recommendations. It is recommended that RH be maintained between 30% and 70% in rooms that house terrestrial species [4]. However, it is possible that RH under 30% will occur, as low temperatures in the external environment and higher altitudes are inherently less capable of holding moisture. Anecdotally, RH at our institution can vary greatly based on season, and recently, a twelve month log of macroenvironmental RH was published from an institution with a similar climate as our own [7]. This raises the question of whether RH in the microenvironment is still higher than in the macroenvironment when the latter is below the minimum recommended level. Further, given the large variation in macroenvironmental RH, it is undocumented if microenvironmental RH follows the same pattern or if it is more stable.

This is of particular concern for our group as we manage a large breeding colony of transgenic cystic fibrosis (CF) mouse models. First, efficient reproduction is a top priority, and low RH has been shown to delay the age at which female mice reach puberty [8]. Second, CF is caused by an autosomal recessive mutation on the *cftr* gene [9], and homozygous animals can display a severe phenotype [10,11,12,13]. Survival in these model animals can be compromised, so if any environmental parameters could be optimized to improve CF mouse survival, it would improve both animal welfare and colony efficiency. Historical data from our colony suggest that around one third of CF pups are lost within the first week of life, so this time period is of particular interest to us. Since seasonal variations in RH are already observed in our facility, this factor was chosen as a starting point.

There were two primary aims for this study. First, we aimed to compare RH in the macroenvironment and microenvironment across various housing configurations and time points. Specifically, microenvironmental RH based on the number of animals in the cage, the time since cage change, and multiple times throughout the year were of interest. Since past work showed that RH is higher in cages of five females than in the macroenvironment, we wanted to determine if this pattern was sustained across different group sizes and when the macroenvironmental RH was below the minimum recommended value of 30%. Second, we wanted to determine if microenvironmental RH was related to pup survival in the first week of life. In general, this is a critical time period for pup survival. Detailed observations of parturition and the neonatal period showed that pups who were found dead stopped moving within the first days after birth [14]. While our ultimate goal is to improve CF mouse survival, further knowledge of how environmental factors impact pup survival in general serves as a stepping stone for future work focused on individual genotype. In addition, knowing how RH is affected by different conditions will help other investigators account for it in various research settings (e.g., in cages of solitary vs. group housed animals or different times of the year).

## 2. Materials and Methods

### 2.1. Animals

All mice used in this study were produced at Case Western Reserve University (Cleveland, OH, USA) on a C57BL/6J background from Jackson Laboratory (Bar Harbor, ME, USA). Cages were taken from a transgenic colony of CF models. Mice homozygous for any mutation have reduced fertility and do not breed well [15,16,17]. Therefore, breeding cages consisted of heterozygous animals which were phenotypically normal. Cages of mice with mutations in modifier genes of CF were also used, but individuals observed here were also phenotypically normal. For a complete list of the strains used, refer to Table S1. All mice were specific pathogen-free (free of mouse parvovirus, mouse hepatitis virus, mouse roto virus, Sendai virus, minute virus of mice, Theiler’s murine encephalomyelitis, pneumonia virus, reovirus, lymphocytic choriomeningitis virus, mouse adenovirus 1 and 2, ectromelia, polyomavirus, mouse cytomegalovirus, *E. cuniculi*, *Filobacterium rodentium*, *Clostridium piliforme,* pin worms, and fur mites) and housed in microisolator cages on individually ventilated racks set at 60 ACH (model MD75JU140MVSP-A, Allentown Inc., Allentown, NJ, USA). Cages contained corn cob bedding (TR Last Co., Saxonburg, PA, USA), breeder chow (Teklad 7904, Envigo, Indianapolis, IN, USA), and acidified drinking water. Mice were given a paper hut (Bio-Serv, Flemington, NJ, USA) and four cotton nestlets (Ancare, Bellmore, NY, USA) for nesting material. Mice were housed under a 12:12 light:dark cycle.

To assess seasonal (summer vs. winter) variation in microenvironmental RH, same sex holding cages and trio breeding cages were used in this study. Same sex cages were taken from the stock of back up breeding mice in the colony. This gave the mice an extra purpose before they were bred or an alternative purpose if they were not ultimately needed for breeding. The mice in the same sex cages were at least 7 weeks of age for collection, and the oldest were 53 weeks of age. A factorial design was used based on sex and group size. Cages either had 1, 3, or 5 mice present. Further, repeated measures were taken from each cage to record how RH changed in relation to time since cage change. Data were collected at 24 h, one week, and two weeks after cage change for each cage. This series was done once per cage. Sample size was determined a priori using Mead’s resource equation [18]. *N* = 60 observations (3 observations from *N* = 20 cages) were needed to have adequate error degrees of freedom. To be balanced across sex and group size, each combination needed at least *n* = 4 cages (*N* = 24 cages). However, this would have equated to *n* = 2 cages per sex, group size, and season. Therefore, we aimed to have *n*= 6 cages per sex and group size (*N* = 36 cages). However, due to availability of *n* = 5 cages only for singly housed males, singly house females, and groups of 5 females, *N* = 33 cages were observed.

For trio breeding cages, the design was based on litter age. Each cage contained a male, two females, and a single litter of pups. Cages were selected for observation if the litter had 5 to 8 pups and if the pups were of the following ages: PD1, PD10, and PD18. Data were also collected from cages with no litter. Again, sample size was determined a priori with Mead’s resource equation and *n* = 12 cages of each litter age were used (*N* = 48 cages). For both same sex cages and breeding cages, half of the cages were observed in the late winter (6 February–6 March) and half in the late summer (15 August–26 September). These time periods were selected to encompass weather extremes in our region.

An additional group of breeding cages was used to observe weekly changes in RH. Once a week for 23 weeks (March to September), a single breeding trio was selected for observation if it contained a single litter of 4 to 8 pups between PD0 and PD2 (*N* = 23). All weekly measurements compared a matched trio of average temperature and RH data points: a breeding cage, the open-air housing room, and a sham cage containing chow, water, and nesting material. Weekly RH data from the breeding cages were also compared to rates of pup loss in our colony. For each measurement week (Sunday to Saturday), the total number of pups born in the room was calculated. The number of pups born each week ranged from 382 to 634 (IQR: 465–546). The presence of a new litter is typically recorded within a day or two of birth. Every Monday to Friday, two members of the lab staff visually inspected the cages that were marked with a pregnant female, so litters were recorded on the day of birth. On the weekends, cages were not checked daily. Consequently, that means that litters born on weekends likely have underestimated pup loss [19]. We acknowledge this short coming, and proceed under the assumption that all of the weekly data points were impacted equally by this. Overall, 22.82% of pups were born on a weekend (2658/11,648), and were most impacted by this shortcoming. On a weekly basis, the percent of pups born on a weekend ranged from 12.61% to 38.35% (IQR: 17.39–25.14%). The number of pups alive at PD6 was also calculated. This age was chosen as this is when tissue samples were collected for genotyping in our colony and recounting pups at this time is standard practice. The proportion of pups alive at PD6 was used as the response variable in the subsequent model described below (Section 2.3.3).

All cages came from the same housing room, taken from eight different racks.

### 2.2. Humidity Collection

**Figure 1:**
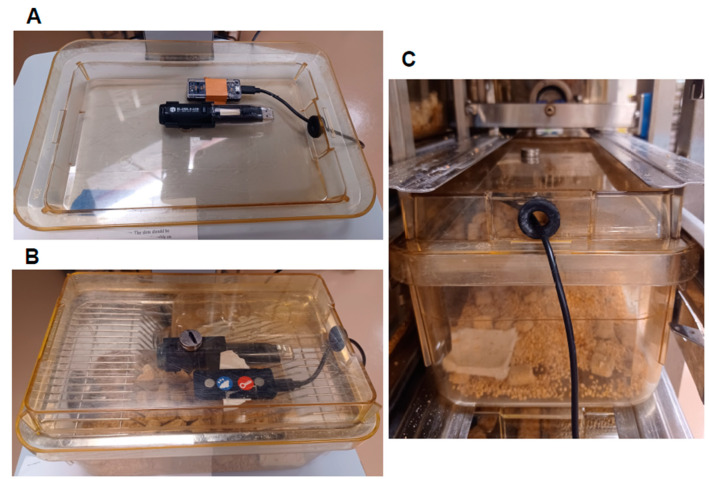
Humidity data logger placement on the cage lid. The corded and cordless data loggers were place adjacent to each other throughout data collection. (**A**) Data loggers are shown with the lid upside down. (**B**) Data loggers are shown with the lid placed on the empty sham cage. (**C**) The sham cage is shown after placement in an IVC rack, with the data logger cord positioned at the front of the cage.

Data were initially collected using two different data loggers in the same breeding cage to assess reliability between two models present in the laboratory. The first was a wireless USB data logger (EZ log, MicroDAQ, Concord, NH, USA) attached to the top of a solid cage lid using magnets. The second was a corded data logger (Yocto-Meteo-V2, Yoctopuce, Geneva, Switzerland) connected to a tablet with Sensory Sentinel software (version 3.2., Turner Scientific, Jacksonville, IL, USA). To accommodate the corded data logger, a small hole was drilled into the side of the cage lid and lined with a rubber grommet. The corded data logger was secured to the cage lid with tape adjacent to the wireless data logger. The data logger placement is shown in Figure 1. Both data loggers remained secured to a single lid that was transferred between the study cages.

Data were collected from each cage for a total of seven minutes. The first two minutes were considered an acclimation period for the data loggers to adjust from air in the room to air in the cage. This was based on pilot testing. It took until after the two minute mark for the standard deviation of RH to be less than 1%. The final five minutes contained data for analysis. Data were collected in order to be minimally disruptive to the mice. During collection, each cage was removed from the rack, placed in a class II A2 fume hood (NU-629-600, Nuaire, Plymouth, MN, USA) to receive the lid with the data loggers, and placed back on the ventilated rack for the seven minute period. Cages were then placed back in the hood to have their original cage lid replaced. Cage order during data collection was randomly assigned using a random number generator (RANDOM.org). It was not possible to be blinded to group size or litter age during data collection, as the mice were visible through the cage wall.

### 2.3. Statistics

All statistics were done using JMP Pro (version 17.0.0).

#### 2.3.1. Data Logger Reliability

The wireless data logger logs data every 10 s and the corded data logger logs data every second. Consequently, data from the corded data logger were filtered to match timepoints from the wireless data logger. Data logger reliability was assessed using the 24 breeding cages monitored in the winter. Due to litter availability, data were collected over three different days, so room level data were also collected on each study day. In total, 832 data points were used to assess temperature and RH reliability. Cronbach’s alpha was calculated for temperature and RH data from each data logger. Temperature reliability was 0.79 and RH reliability was 0.98 between the two data loggers. While RH reliability was excellent between the two data loggers, all subsequent analyses were based on data with the corded data logger as it logged more sensitive temperature data based on a plot of the raw data (Figure S1).

#### 2.3.2. Seasonal Variation

Breeding cages and same sex cages were analyzed separately. For each cage, the average temperature and RH were calculated for each five minute recording period. All temperature and RH data were analyzed using residual maximum likelihood mixed models. Temperature and humidity were the response variables, each in their own model. Data were analyzed in two steps. First, data were compared between the room and the home cage. Due to animal availability, home cage data were collected across multiple days, so data from the room were collected in the open air for a five minute period on each day that data were collected from animal cages. For models comparing data from the home cage and room, the collection location, season (winter vs. summer), and the interaction were tested as fixed effects. Collection date was included as a random factor. CageID was also included as a random factor in the same sex models due to the repeated measures from each cage.

Then, data from the home cage only were analyzed. Temperature and humidity were analyzed again as response variables in their own models, since the factors of interest in the home cage could not be assigned to the room in the previous models. For breeding cages, litter age, season, and the interaction were tested as fixed effects, and collection date was included as a random factor. For same sex cages, group size, sex, season, and time since cage change were tested as main effects. All two-way interactions were included and dropped if insignificant. Three-way interactions were not possible to analyze as there were only *n* = 2 cages of singly housed males, singly housed females, and groups of 5 females recorded in the winter. To analyze two-way interactions, all groups had at least *n* = 5 cages. Collection date and CageID were included as random factors. Any significant main effects were further analyzed with post hoc contrasts (*p* < 0.05). Multi-collinearity between the predictors was assessed using variance inflation factor (VIF). The VIF values ranged from 1.00 to 2.29 between the predictors across all models. All model assumptions were assessed post hoc by assessing the normal Q-Q and residual vs. predicted plots. Data transformations were made as needed to meet model assumptions and are indicated in the appropriate sections. A log10 transformation was needed for the temperature data in the breeding cages to meet model assumptions and a square root transformation was needed for the RH data to meet model assumptions when comparing RH from the same sex cages and the room.

#### 2.3.3. Spring to Fall Data

First, temperature and RH data were compared between the breeding cage, room, and sham cage using a residual maximum likelihood mixed model. Here, temperature and RH were analyzed as response variables in their own models again. The collection location, external temperature at the start of measurements, and the interaction were tested as fixed effects. The VIF values ranged from 1.00 to 1.33 between the predictors across both models. Weather data were based on the NOAA station in Cleveland, Ohio (41.5° N, 81.7° W). Collection date was included as a random effect.

Second, temperature and RH data were tested for an impact on weekly pup loss, based on survival at PD6. Pup loss was the response variable. Pearson’s correlation coefficient was calculated between weekly pup loss proportion, average cage RH and temperature, average room RH and temperature, and the external temperature. Finally, a regression model was run to test the effect of average cage RH and temperature on weekly pup loss proportion. The correlation calculations were used to identify potential predictors that may be related to pup loss and to prevent the simultaneous analysis of any predictors that may be correlated with each other. All model assumptions were assessed post hoc by assessing the normal Q-Q and residual vs. predicted plots.

## 3. Results

### 3.1. Seasonal Variation

Home cage temperature was warmer than room temperature for both breeding cages (F_1,47.25_ = 11.22, *p* = 0.002) and same sex cages (F_1,55.10_ = 29.78, *p* < 0.001). The predicted means for each measurement location are shown in Table 1. When assessing factors present in the home cage only, there were no significant effects on home cage temperature for either breeding cages or same sex cages (*p* > 0.05).

**Figure 2:**
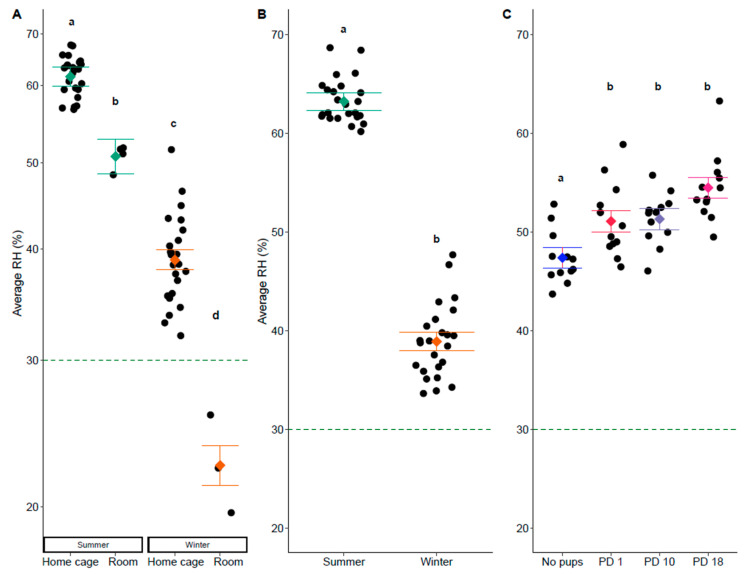
Average relative humidity in the breeding cage and room. (**A**) Humidity was impacted by an interaction between season and measurement location (*p* < 0.001). (**B**) In breeding cages only, humidity was impacted by (**B**) season (*p* < 0.001) and (**C**) the presence of a litter (*p* < 0.001). All panels show the minimum humidity levels recommended by the guide as a dashed line at 30%, and the factor level LSM ± SE over a scatter of the individual predicted + residual data points. The *Y*-axis for panel A is show on a log10 back-transformed scale. Different letters in the panels represent statistically significant differences.

Home cage RH was impacted by several factors in both breeding and same sex cages. In breeding cages, there was an interaction between season and measurement location (F_1,46.69_ = 23.22, *p* < 0.001). RH in both the room and the breeding cage was higher in the summer than the winter, and RH was higher in the breeding cage than in the room across seasons (Tukey: *p* < 0.05, Figure 2A. Table S2). When looking at breeding cage measurements only, RH was impacted by season (F_1,5.03_ = 352.69, *p* < 0.001) and litter presence (F_3,38.82_ = 10.18, *p* < 0.001). Home cage RH was higher in the summer (Figure 2B, Table S3), and it was higher in cages that had any litter present than in cages without a litter (Tukey: *p* < 0.05, Figure 2C, Table S3).

**Figure 3:**
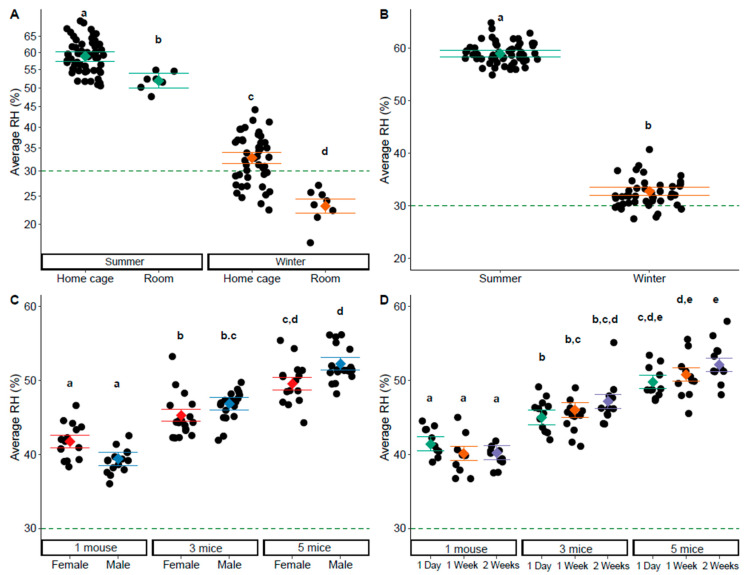
Average relative humidity in same sex cages and the room. (**A**) Humidity was impacted by an interaction between season and measurement location (*p* = 0.036). (**B**) In holding cages only, humidity was impacted by (**B**) season (*p* < 0.001), (**C**) an interaction between sex and group size (*p* = 0.006), and (**D**) and interaction between group size and the time since cage change (*p* = 0.042). All panels show the minimum humidity levels recommended by the guide as a dashed line at 30%, and the factor level LSM ± SE over a scatter of the individual predicted + residual data points. The *Y*-axis for panel (**A**) is show on a square root back-transformed scale. Different letters in the panels represent statistically significant differences.

In same sex cages, there was an interaction between season and measurement location (F_1,50.01_ = 4.64, *p* = 0.036). Home cage and room RH was higher in the summer than the winter, and RH was higher in the holding cage than in the room across seasons (Tukey: *p* < 0.05, Figure 3A, Table S4). When looking at home cage measurements only, RH was impacted by season (F_1,13.40_ = 676.60, *p* < 0.001), an interaction between sex and group size (F_2,26.02_ = 6.27, *p* = 0.006), and an interaction between group size and the time since cage change (F_4,59.29_ = 2.65, *p* = 0.042). Home cage RH was higher in the summer (Figure 3B, Table S5). While there were two significant interactions involving group size, post hoc tests pulled out effects of group size alone. In both female and male cages, home cage RH increased with group size (Tukey: *p* < 0.05, Figure 3C, Table S5). At each time point post cage change, home cage RH increased with group size (Tukey: *p* < 0.05, Figure 3D, Table S5).

### 3.2. Spring to Fall Data

Weekly data recorded between March and September showed that the temperature in the animal facility was impacted by collection location (F_2,40.60_ = 7.38, *p* = 0.002) and the external temperature (F_1,20.10_ = 5.46, *p* = 0.030). Temperature was higher in the breeding cage (LSM: 23.30 ± 0.09 °C) than in the sham cage (LSM: 22.92 ± 0.09 °C) or the housing room (LSM: 22.97 ± 0.09 °C, Tukey: *p* < 0.05). As outside temperature increased, temperature in the facility decreased, regardless of if the data logger was in a cage, or in the open room air (coefficient = −0.012 ± 0.005).

RH in the animal facility was also impacted by collection location (F_2,41.08_ = 103.43, *p* < 0.001) and the external temperature (F_1,20.94_ = 48.76, *p* < 0.001). RH was higher in the breeding cage (LSM: 53.71 ± 1.48%) than in the sham cage (LSM: 40.79 ± 1.49%) or the housing room (LSM: 41.65 ± 1.48%, Tukey: *p* < 0.05). As outside temperature increased, RH in the facility increased, regardless of if the data logger was in a cage, or in the open room air (coefficient = 0.774 ± 0.111).

**Figure 4:**
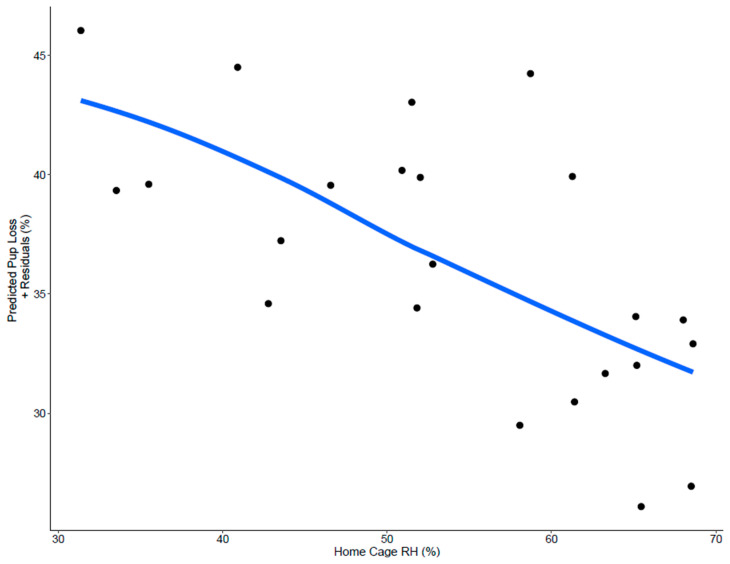
The weekly percent of pup loss over 23 weeks was impacted by average humidity in the breeding cage (*p* = 0.003). The figure depicts the best fit line over a scatter of predicted + residual data points.

Weekly pup loss, average cage level RH, average room level RH, and external temperature were significantly correlated (Table 2). Based on these findings, it was not possible to test all of the latter three variables for an effect on pup loss due to potential multicollinearity issues. Therefore, it was decided to only include cage level RH in the model, since the mice have more direct exposure to this parameter. A linear regression model showed that average cage level RH impacted pup loss (F_1,20_ = 11.65, η^2^_p_ = 0.31, *p* = 0.003). As cage level RH increased, the proportion of pups lost each week decreased (coefficient = −0.299 ± 0.088, Figure 4).

## 4. Discussion

These data show that RH is quite variable between the macroenvironment and microenvironment of laboratory mouse housing rooms. Compared to the room level readings, breeding cages had a predicted average RH value 11.81% higher during the summer, and 16.51% higher during the winter. Similarly, same sex cages had a predicted average RH value 6.85% higher during the summer and 9.69% higher in the winter than the housing room. These differences were present despite relatively consistent temperature readings. While statistically significant differences in temperature were found between the cages and the room, predicted values differed by less than 1 °C, making the variation in temperature proportionally much less than that of RH. This calls into question the current practice of recording room level parameters when IVC racks are present in a rodent housing room. Macroenvironmental readings should not be assumed to indicate microenvironmental readings. While this practice may be appropriate for some parameters (i.e., temperature), it does not accurately reflect others (i.e., RH). Our findings show a similar pattern as past work [5,6], and suggest a need for microenvironment measurements to be assessed to more accurately reflect current housing conditions.

RH is also variable within cages of the same housing room based on time of year and cage population. Despite efforts to standardize the environment of laboratory animals, these and past data [7] indicate that more advances are needed in RH control to overcome seasonal variations. Comparisons between summer and winter data revealed a 24.34% and 26% predicted difference in breeding and same sex cage level RH values, respectively. This could act as a confounding variable during longitudinal research and potentially hinder efforts to improve reproducibility. While research focused on biological impacts of RH in laboratory mice is lacking, it is known that ammonia build-up in the cage, and pathogen stability and transmission, are impacted by RH: ammonia build-up occurs at higher RH, and the influenza virus is spread more easily at low RH [20,21,22]. While mice are not natural hosts of the influenza virus, it is unknown if viruses that they are susceptible to behave in a similar way.

Group size also impacted RH in all observed cages: RH increased as the number of adult animals increased, and with the presence of a litter. This is supported by past research [23] and emphasizes the importance of including multiple group sizes in studies where measurements could be impacted by RH in the cage. Although the interaction between group size and season was not significant, it is worth noting the variation observed in same sex cages during the winter. Figure 2B shows that a subset of data points fall below the minimum recommended RH value of 30%. In terms of the raw data, 15 observations were under 30%, with 10 of those corresponding to cages of singly housed mice in the winter. This observation raises concern for singly housed animals in the winter. These animals may be living in chronically low cage level RH. It is known that RH under 30% can lead to ocular irritation, is more favorable for the spread and stability of viruses, and can initiate the development of ringtail [20,21,24,25]. Previous research on human subjects could aid in this field through a reverse translational lens. In comparison to 50% RH baseline, humans exposed to 30% RH had increased frequency of blinking, and greater sensations of dryness in their nose and throat that did not subside after 90 min of exposure [26]. While this does not reflect the continuous exposure that the mice experience, the true scope of how much RH variation occurs in IVC housing remains unknown. Further research is warranted to document hourly or even minute-by-minute changes that the animals could experience, and how their physiology may be affected.

Weekly data taken between March and September showed that RH inside the housing room and IVC mouse cages is strongly correlated with the temperature of the external environment, despite relatively stable temperatures in the cage. This in turn was associated with the number of mouse pups that died in the first week of life. As a whole, the house mouse is known to be an opportunistic breeder that does not necessarily depend on seasonal conditions to reproduce [27]. However, inbred laboratory strains have variable breeding metrics, and maternal behavior has a strong influence on litter survival [28,29,30]. It is often advised to keep disturbances to a minimum for pregnant dams [31]. The disadvantage of this practice is that if mothers with litters are not disturbed, pups at birth cannot be counted to compare to pups at weaning. In fact, it is known that most pup loss occurs in the first two days after birth [19]. This leaves many studies using a crude metric of total litter loss, which might not be sensitive enough to pick up any environmental signal that impacts pup survival. In this study, pup survival was collected between PD6 and PD8, which allowed us to quantify individual pup survival instead of total litter loss. However, pup loss overall may still have been underestimated, as daily checks are not as sensitive as video data in estimating litter size and loss [19]. Our group unfortunately did not have the resources to collect video data, but it is an area of improvement for future work.

To the best of our knowledge, this is the first report of pup survival being impacted by RH. This is not to suggest that RH impacts reproduction to the same extent as strain or maternal behavior. However, the use of a more sensitive measure allowed us to identify this environmental factor as a potential influencer of reproductive success. Ultimately, this could be a crucial factor for producing sensitive disease models that are not fertile themselves and must be produced through heterozygous mating schemes. In our case, we cannot yet confirm if CF pup survival specifically is related to RH. The pup carcasses are typically cannibalized by the mothers before we are able to find and genotype them. However, this is a priority for future work.

Generally speaking, further research is needed to confirm a cause and effect relationship based on the current association between RH and pup survival. The use of controlled RH conditions is essential in order to gauge the full extent of this parameter’s biological impact. The data presented here are meant to bring awareness to the variation that occurs in an environmental factor that is often recorded, but not often considered for its potential impact on terrestrial animal models. While it is standard practice to record RH in the macroenvironment, we have shown that this does not reflect the laboratory mouse’s microenvironment. However, this work could be expanded, since RH is known to be further impacted by the air changes per hour (ACH) of IVC racks and bedding volume [5,23]. The racks here were all set to 60 ACH and this study used a single bedding type, corn cob, which is allocated at roughly 100 g per cage. While the majority of these data were collected with a costly monitoring system, our sensor reliability calculations showed that an inexpensive USB data logger is capable of producing similar data. We do acknowledge a limitation in that the data loggers were located on the cage lid instead of being level with the mice. This was done to prevent the mice from contacting and damaging the data loggers. Future methods would ideally develop a way to mount the data loggers at the animal level, allowing sufficient air circulation while preventing direct contact. Additionally, while we observed distinct differences between RH in the home cage and RH in the room, it was not possible to achieve an airtight seal in the cage lid due to the hole that was required for the cord. This permitted air from the cage to escape into the room based on the positive pressure IVC racks that were used. The wireless data logger would have permitted an airtight seal, but given the difference in temperature sensitivity between it and the corded sensor, we opted for the latter. Nonetheless, it is our hope that more researchers will consider how variation in RH could impact their animal model and the data that are collected from them.

## 5. Conclusions

In conclusion, this study shows that RH is not always well controlled in vivaria, displaying seasonal variation. It also shows that the standard practice of measuring RH at the room level is not an accurate representation of what laboratory mice experience inside of ventilated cages. RH may act as an extrinsic factor impacting animal welfare. Here, it showed an inverse correlation with pup survival. This work was meant to highlight the need for additional research aimed at understanding the full scope of RH’s impact on laboratory mice. Researchers should be aware of RH patterns in their facility, and keep it in mind for potential impacts on animal welfare and reproducibility.

## Figures and Tables

**Table 1:** Predicted temperature means (°C) in the animal housing room and holding cages. Data transformation method is listed where appropriate, and any relevant back transformed values are presented in parentheses. “---” indicates a value is not applicable.

Measurement Location	Transformation	Predicted Mean	Standard Error
Breeding cages			
Home cage	log10	1.389 (24.55)	0.001
Room	log10	1.378 (23.99)	0.003
Same sex cages			
Home cage	---	24.138	0.095
Room	---	23.298	0.142

**Table 2:** Pearson’s correlation coefficients for weekly proportion of pup loss, average temperature and humidity in the breeding cage and housing room, and the outside temperature recorded at the start of data collection. Values in bold were significant at *p* < 0.01.

	Weekly Pup Loss	Cage RH (%)	Room RH (%)	Cage Temp (F)	Room Temp (F)	Outside Temp (F)
Weekly Pup Loss	1.0000	−0.6682	−0.5742	0.3692	0.4388	−0.5727
Cage RH (%)		1.0000	0.9304	−0.4341	−0.2934	0.8049
Room RH (%)			1.0000	−0.3684	−0.1833	0.8198
Cage Temp (F)				1.0000	0.7136	−0.2707
Room Temp (F)					1.0000	−0.2291
Outside Temp (F)						1.0000

## Data Availability

Data are contained within the article or Supplementary Material.

## Supplementary Materials

The following supporting information can be downloaded at: https://www.mdpi.com/article/10.3390/ani14223164/s1, Figure S1: raw temperature and relative humidity data; Table S1: list of strain nomenclature from cages used to collect home cage humidity readings; Table S2: median, minimum, and maximum RH values across breeding cages and in the room; Table S3: median, minimum, and maximum RH values across breeding cages only; Table S4: median, minimum, and maximum RH values across same sex cages and in the room; Table S5: median, minimum, and maximum RH values across same sex cages only; raw data tables.
