# Supplementary material for: Think Beyond the Room: Measuring Relative Humidity in the Home Cage and Its Impact on Reproduction in Laboratory Mice, Mus musculus
Source: Animals (Basel). 2024 Nov 5;14(22):3164. doi: 10.3390/ani14223164 (PMC11591041; doi:10.3390/ani14223164)
Supplement: Supplementary file 1 [file animals-14-03164-s001.zip › SupplementaryInformation20241021.pdf]

Table S1. List of strain nomenclature from cages used to collect home cage humidity readings.

Internal Strain Label	Formal nomenclature	Count of breeding cages	Count of same sex cages
A14	C57BL/6N-*Slc6a14tm1a(KOMP)Wtsi*/TcpMmucd	3	1
AI	B6.Cg-Gt(ROSA)26Sor$^{tm9(CAG-tdTomato)Hze}$/J		1
B6	C57BL/6J	1	12
BC	B6.129-*Cftr*	1	
CB	C57BL/6-*Crbn*$^{tm1.1Ble}$/J		2
CBX	B6.Cftrem3Cwr x C57BL/6-*Crbn*$^{tm1.1Ble}$/J	3	
CDEL	B6.Cftr^{em17Cwr}	2	
CDF	B6.Cftr^{em1Cwr}	1	
CGD	B6.Cftr^{em4Cwr}	1	1
CGX	B6.Cftr^{em3Cwr}	9	2
CI	B6.Cftr^{em2Cwr}		1
CRV	B6.SJL-GT(ROSA)26Sorem1(CAG-mVen,-Akluc) x B6.C-Tg(CMV-cre)1Cgn/J	2	1
CWX	B6.Cftr^{em5Cwr}	2	
D	B6.129S6-*Cftr*^{tm1Kth}/J	4	
E11	B6.Cftr^{em6Cwr}	1	
E12	B6.Cftr^{em7Cwr}	1	
E3	B6.Cftr^{em13Cwr}	3	
EG2	B6.Cftr^{em8Cwr}	4	1
ER	B6.Cftr^{em9Cwr}	1	3
FD	Cftr^{tm1Kth}Tg(FABPCFTR)1Jaw/Cwr	3	2
FR	B6.SJL-GT(ROSA)26Sor$^{em1(CAG-mVen,-Akluc)}$	1	
GR	B6.SJL-GT(ROSA)26Sor$^{em1(CAG-CFTR*G542X,-mVen,-Akluc)}$	1	
GSNOR	B6.129-GSNOR^{tm1Llli}	2	
HCT	B6-*Cftr*^{em2Cwr} Tg(CFTR$^{3849+10kbC>T}$)	2	
HDFT	B6-Tg(*Cftr*^{F508del})Cwr	1	
HE	Tg(CFTR)1Lcl/Cwr	2	
HGD1	B6-*Cftr*^{em2Cwr} Tg(CFTR^{G551D})Cwr	2	
HMC	B6.Cftr^{em2Cwr} Tg(CFTR)1Lcl/Cwr	1	
hN	B6.Cftr^{em2Cwr} Tg(CFTR^{I507del})1Lcl/Cwr	1	
hN	B6.Cftrem2Cwr Tg(CFTR)1Lcl/Cwr	2	

hWX	B6-*Cftr*^{em2Cwr} Tg(CFTR^{W1282X})Cwr	2	
KC	B6.SJL-Kcnab2^{em1Cwr}	1	
KCV	B6.SJL-Kcnab2em1Cwr x B6.C-Tg(CMV-cre)1Cgn/J	1	
MT	B6.129(Cg)-*Gt(ROSA)26Sor*$^{tm4(ACTB-tdTomato,-EGFP)Luo}$/J	1	
N	B6.129S6-Cftr^{tm1Mrc}	1	
NPF	C57BL/6N-Npr3^{tm1a}(KOMP)Mbp/Mmucd x B6.Cg-Tg(Pgk1-flpo)10Sykr/J	1	
NPM	C57BL/6N-Npr3^{tm1a}(KOMP)Mbp/Mmucd x B6.C-Tg(CMV-cre)1Cgn/J	1	1
NPR	C57BL/6N-Npr3^{tm1a}(KOMP)Mbp/Mmucd	1	
RR	B6.SJL-GT(ROSA)26Sor$^{em1(CAG-CFTR*R553X,-mVen,-Akluc)}$		1
S	B6.129P2-*Cftr*^{tm1Unc}/J		1
SFCG	B6.129-GSNORtm1Llli X B6.129S-*Sftpc*$^{tm1(cre/ERT2)Blh}$/J X B6N.129S6(Cg)-Scgb1a1tm1(cre/ERT)Blh/J	2	
T2CIC	B6.Cftr^{tm2Cwr} X B6.129-Gt(ROSA)26Sor$^{tm1(cre/ERT2)Tyj}$/J	1	
TP	C57BL/6N-*Tppp*$^{tm1.1(KOMP)Vlcg}$/JMmucd	1	1
WB	B6.Tyr^{em1Cwr}		1
WR	B6.SJL-GT(ROSA)26Sor$^{em1(CAG-CFTR*W1282X,-mVen,-Akluc)}$		1
WTR	B6.SJL-GT(ROSA)26Sor$^{em1(CAG-CFTR,-mVen,-Akluc)}$	1	

Table S2. S2 Table. Median, minimum, and maximum of the average RH values recorded in the breeding cages and in the room. Values were calculated from data that were collected every ten seconds during a five minute recording period and averaged per cage or room.

Factor	Median RH (%)	Minimum RH (%)	Maximum RH (%)
Summer*	63.04580613	48.52999973	68.7064511
Winter*	38.1266668	19.62903226	51.91333373
Home Cage#	54.86666725	31.54000003	68.7064511
Room#	48.52999973	19.62903226	52.16333323

*calculated across all breeding cages and room data
#calculated across both seasons

Table S3. Median, minimum, and maximum of the average RH values recorded in the breeding cages only. Values were calculated from data that were collected every ten seconds during a five minute recording period and averaged per breeding cage.

Factor	Median RH (%)	Minimum RH (%)	Maximum RH (%)
Summer*	64.39666738	57.82000077	68.7064511
Winter*	38.68499988	31.54000003	51.91333373
No pups#	48.52612943	31.54000003	64.97333273

PD 1#	53.70666695	34.02333353	68.7064511
PD 10#	51.99777746	33.30645145	66.9000005
PD 18#	58.15500047	38.1266668	68.55333327

*calculated across all breeding cages and room data
#calculated across both seasons

Table S4. Median, minimum, and maximum of the average RH values recorded in the same sex cages and in the room. Values were calculated from data that were collected every ten seconds during a five minute recording period and averaged per cage or room.

Factor	Median RH (%)	Minimum RH (%)	Maximum RH (%)
Summer*	58.47333353	47.67666663	69.71333337
Winter*	31.39032255	16.92333303	44.27666663
Home Cage#	51.77333337	22.46333323	69.71333337
Room#	27.15333353	16.92333303	54.92258042

*calculated across all same sex cages and room data
#calculated across both seasons

Table S5. Median, minimum, and maximum RH values across same sex cages only. Values were calculated from data that were collected every ten seconds during a five minute recording period and averaged per same sex cage.

Factor	Median RH (%)	Minimum RH (%)	Maximum RH (%)
Summer*	59.13499963	50.5566672	69.71333337
Winter*	32.96333307	22.46333323	44.27666663
1 female§	54.39999983	24.75999987	58.09354845
3 females§	47.74978488	29.0466667	59.6599998
5 females§	59.25666653	31.04333357	69.71333337
1 male§	50.90666703	22.46333323	56.43333333
3 males§	47.85833333	28.74999997	61.75333327
5 males§	53.38666643	36.33333333	69.1133339
1 mouse, 1 day after cage change#	54.40499965	25.27666693	58.09354845
1 mouse, 1 week after cage change#	51.16500028	22.46333323	57.1866666
1 mouse, 2 weeks after cage change#	51.12333353	25.61666673	56.04999967
3 mice, 1 day after cage change#	44.75333302	29.0466667	61.74333363
3 mice, 1 week after cage change#	45.78000015	28.74999997	60.1266668
3 mice, 2 weeks after cage change#	48.83811828	30.20645135	61.75333327
5 mice, 1 day after cage change#	59.01333273	34.3700001	67.06000043
5 mice, 1 week after cage change#	59.67666713	31.04333357	69.1133339
5 mice, 2 weeks after cage change#	62.49666723	38.7933333	69.71333337

*calculated across all same sex cages and room data
§calculated across both seasons and cage change time points
#calculated across both seasons and sexes

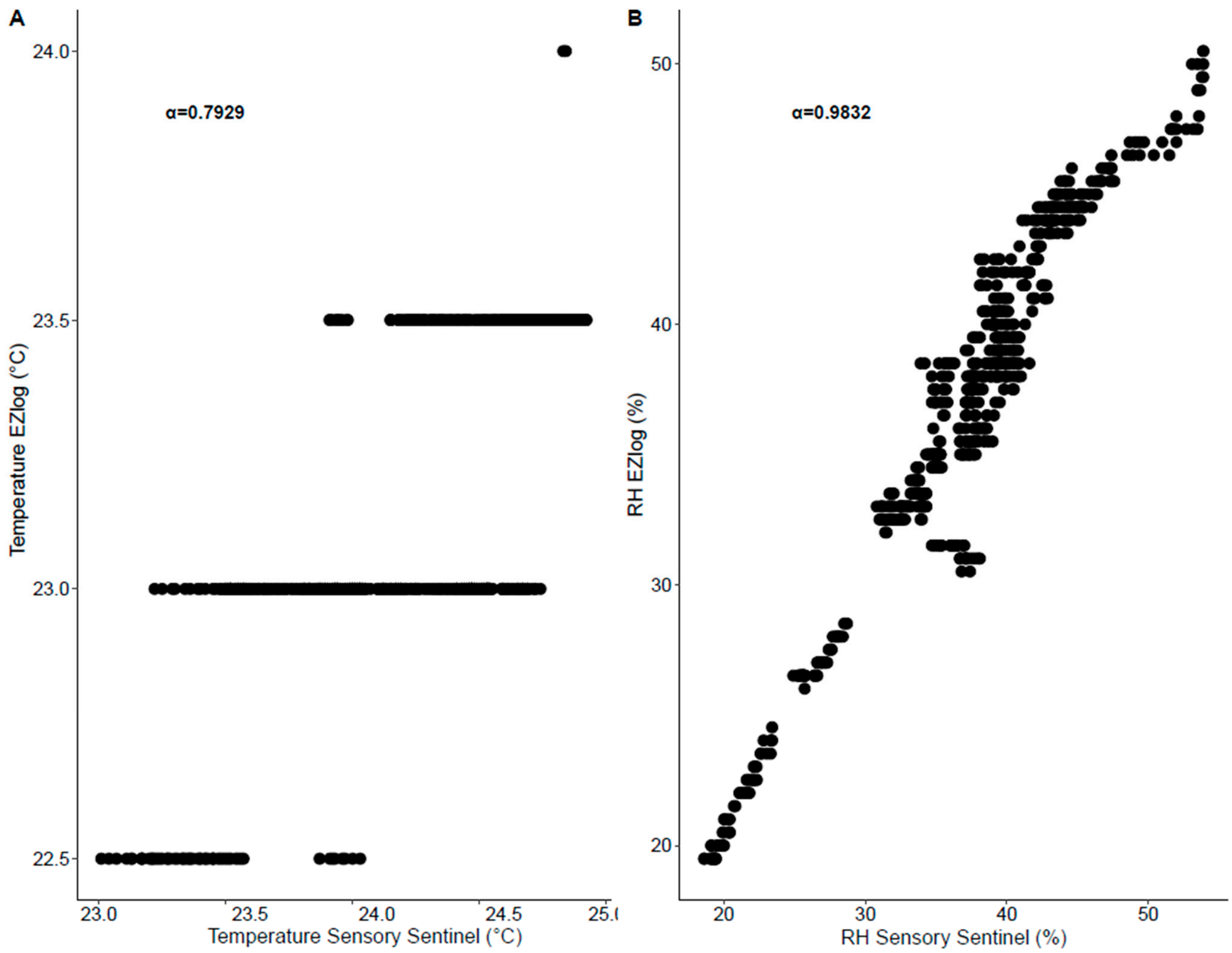

Figure S1. Raw (A) temperature and (B) relative humidity data collected with the corded sensor attached to the Sensory Sentinel package plotted against the data collected from the wireless, EZlog sensor. Data were collected during the winter months and includes those from both the microenvironment and macroenvironment (N=832). Reliability calculated using Cronbach's α is presented on each panel.
